# Registration: A Plea for Unity

**Published:** 1912-05-11

**Authors:** Sydney Holland

**Affiliations:** Kneesworth Hall, Royston, Herts


*To the Editor of* The Hospital.

### Registration: A Plea for Unity.

*To the Editor of* The Hospital.

Sir,—I observe that Mrs. Bedford Fenwick's nursing paper declares in a leading article, presumably from her own pen, that "the opposition to the registration of nurses is narrowed down to a few autocratic governors and their reactionary officers—medical, nursing, and secretarial."

What a charming delusion! Lest it should deceive anyone, please let me say that the protest against the Registration Bill is signed by no less than 244 matrons of hospitals, sixty-six of whom are matrons of London hospitals, or were at the time of signing, and by 1,332 nurses. We have not sought to add a single name since

1909, but none of those who signed, as far as I know, have changed their opinion.

These ladies, who signed the protest, conceivably know their business as well as the ex-matrons and foreigners who compose so active a contingent amongst the agitators for registration. There is certainly no "narrowing down" amongst those who oppose registration, and it strikes me that there is precious little new blood amongst the agitators, if I may judge from the constant recurrence of the same names as reading papers on the subject, and as holding the multitudinous posts in the various societies composed always of the same set of ladies.

The whole of this disagreement amongst us all might be ended by compromise. That is the pity of it. What a good thing it would be if we could all meet on one platform for the good of nurses and nursing, and not have this bugbear of registration separating us. Roughly speaking, all that the registrationists want, or, at any rate, the main thing they want, is to prevent a woman who is not a trained nurse posing as such. If there were an "Official Directory of Nurses," in which every woman nursing for money payment was obliged to be entered and have her training faithfully recorded, then every employer of a nurse could satisfy himself as to the training of the nurse he was employing, and there would be no false guarantee that a nurse was a good and fit nurse because she appeared in the directory. The directory would merely give her training. This is not ideal from the registrationist point of view, but it is a compromise and a good step in advance, and one in which all the anti-registrationists would join. We should see all the nursing world combined, and the millennium, if not reached, would be within measurable distance. At any rate, all these miserable differences would be ended, and that would be something. I do not think that registrationists realise, perhaps, the difficulty of getting a Bill through Parliament to which there is very strong objection. I have little hope of the compromise being accepted, but, if it were, I would work loyally to get it passed.—Yours faithfully, Sydney Holland.

Kneesworth Hall, Royston, Herts,
May 5.

